# An Unusual Presentation of Spigelian Hernia Incarceration after Colonoscopy

**DOI:** 10.7759/cureus.3317

**Published:** 2018-09-17

**Authors:** Vincent M Pronesti, Clara Antoury, Ricardo Mitre

**Affiliations:** 1 Department of Internal Medicine, Allegheny Health Network, Pittsburgh, USA; 2 Department of Gastroenterology, Allegheny Health Network, Pittsburgh, USA

**Keywords:** spigelian hernia, colonoscopy, systemic inflammatory response syndrome (sirs), bowel incarceration, colonic resection, left ventricular assist device

## Abstract

Spigelian hernias are uncommon and predominantly affect the abdominal wall.The incidence of Spigelian hernias after colonoscopy is even rarer with only one case outlined in the surgical literature. This is the case of a 66-year-old man who underwent routine colonoscopy and presented to the hospital with systemic inflammatory response syndrome (SIRS). A computed tomography (CT) scan demonstrated a Spigelian hernia in the location of a prior left ventricular assist device (LVAD) placement. This required surgical resection and resulted in a complicated post-operative course. This case offers a unique perspective on a rare colonoscopic complication not well represented in the literature. It offers the learning point of remaining vigilant for a rare, but potentially deadly, colonoscopic outcome. This case also illustrates the decision-making heuristic of availability bias.

## Introduction

Clinicians must be aware of potential rare complications after colonoscopy. This is particularly relevant because every patient is advised to get a screening colonoscopy at age 50, making this an exceedingly common procedure.

Spigelian hernias are rare and comprise approximately 0.12% of hernias of the abdominal wall [1]. Such hernias are susceptible to incarceration because they are difficult to reduce. The risk of developing such a hernia is increasing as more patients have abdominal wall surgeries [2]. There is only one case in the literature by Demuro which discusses a Spigelian hernia after colonoscopy, although Persinger and Basson recently published a case report discussing the inability to conduct a colonoscopy due to the presence of a Spigelian hernia [3,4]. This is the first case to discuss a Spigelian hernia developing at the site of prior left ventricular assist device (LVAD) placement. It was originally presented in poster format, but has been greatly expanded for the current work (Poster Presentation: Pronesti VM, Antoury C, and Mitre R. An Unusual Presentation of Spigelian Hernia Incarceration After Colonoscopy. American College of Gastroenterology Annual Meeting; October 15, 2018).

This case discussion of a 66-year-old man who underwent routine colonoscopy, returning with worsening abdominal pain and systemic inflammatory response syndrome (SIRS) is a common scenario. This common presentation is what emphasizes the importance of this case. The primary teaching point of this case report is to expand on the heuristic of availability bias in medicine. Ruling out common things first without considering alternate etiologies is a hallmark of availability bias and will often lead to delayed diagnosis and management. This case demonstrates an example of how to avoid availability bias, which is generally seen when a rare diagnosis is excluded from thought processing initially as other more common etiologies are entertained.

## Case presentation

**Figure 1:**
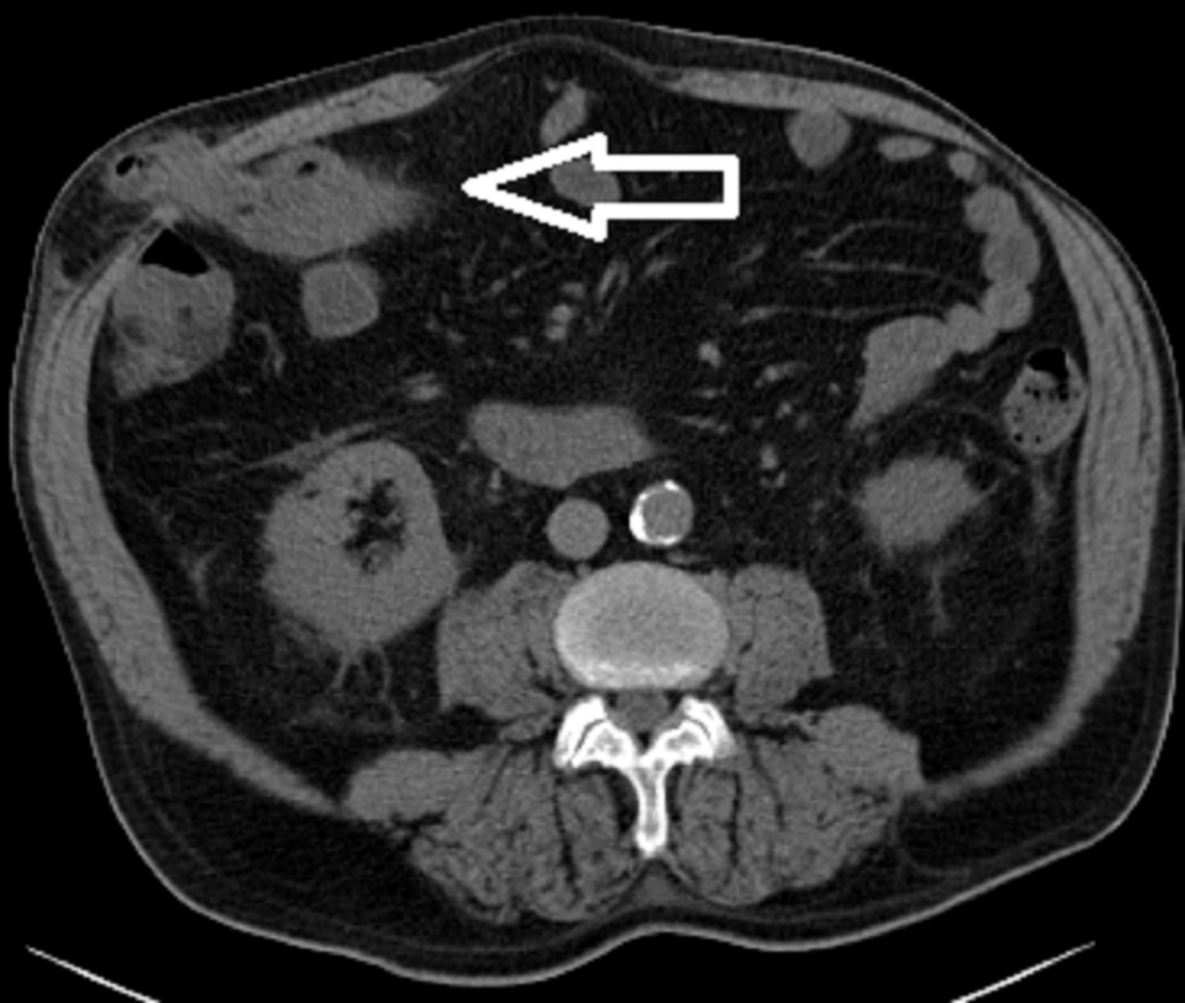
Abdominal wall Spigelian hernia. Computed tomography (CT) scan abdomen showing colonic extension from the hepatic flexure back inferolaterally into a Spigelian hernia with mild fat stranding. The white arrow shows the hernia penetrating through the abdominal wall.

**Figure 2:**
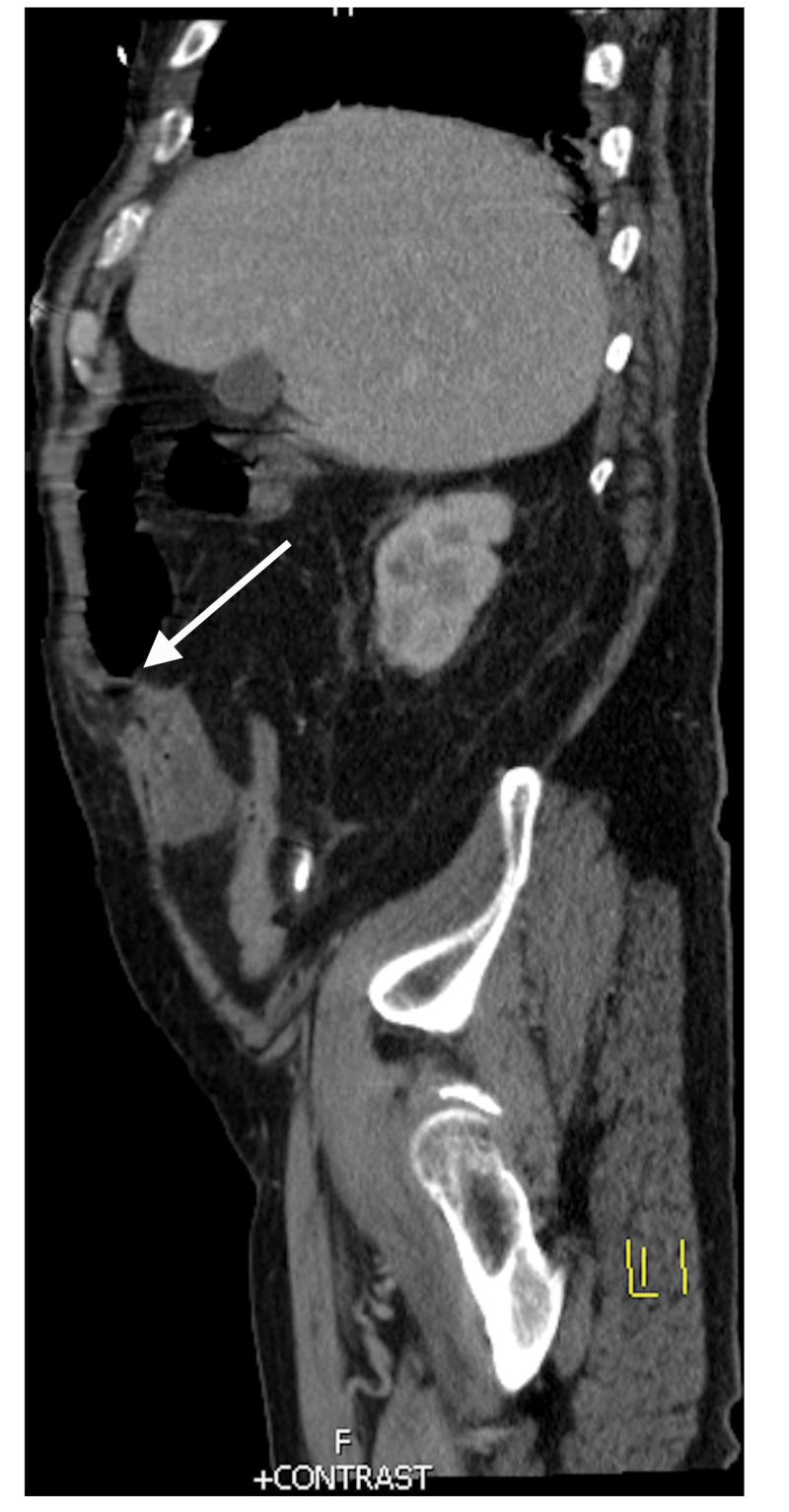
Sagittal view of the Spigelian hernia. The white arrow shows the Spigelian hernia exiting the abdomen through an LVAD site. LVAD: Left ventricular assist device

A 66-year-old man with a past medical history of heart transplant with coronary artery disease of a transplanted heart with prior LVAD placement presented to the gastroenterologist with one year of diarrhea and a reported history of colonic polyps. He complained of occasional stools with blood. Colonoscopy revealed a 12 mm soft, sub-epithelial lesion likely representing a lipoma. Also seen during the procedure were multiple angioectasias and general edema with an atrophic ileum. After the colonoscopy, he developed subjective fevers, chills, non-bloody emesis, and diarrhea. The next day he had abdominal pain and distension. He presented to the hospital, where he was found to be in SIRS with tachycardia and labs significant for leukocytosis of approximately 15000. The diagnosis of microperforation after colonoscopic insufflation was considered, and computed tomography (CT) of the abdomen was obtained to assess for free air in the abdomen to suggest perforation. This scan, seen in Figure 1, did not show any signs of perforation, but did show colonic extension from the hepatic flexure back inferolaterally into a Spigelian hernia with mild fat stranding. It also showed wall thickening of the proximal transverse colon as it exited the hernia. This hernia was occurring through the site of the prior LVAD. Figure 2 illustrates the hernia in a sagittal view and more easily visualizes the herniation through a prior LVAD site.

**Figure 3:**
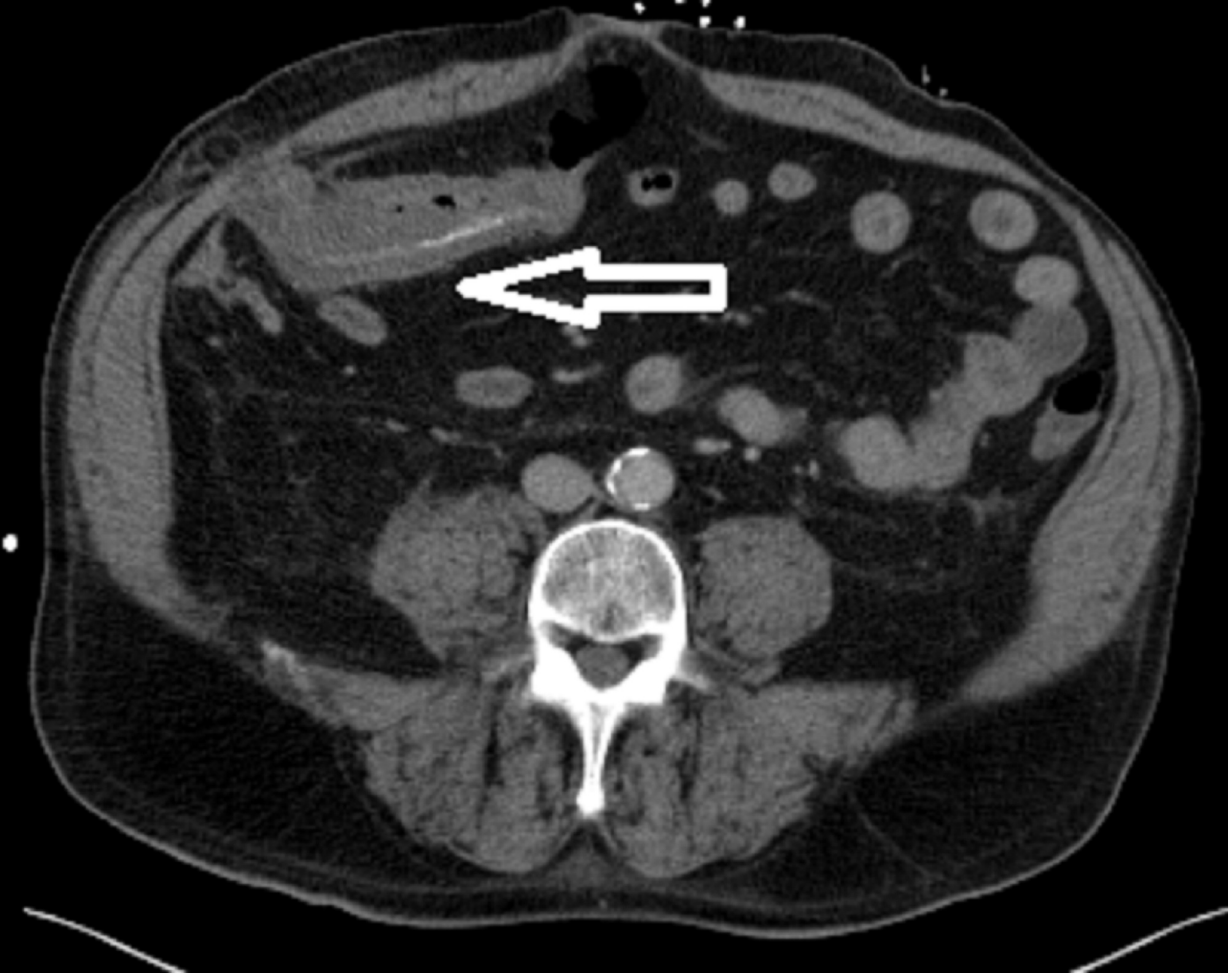
Follow-up computed tomography (CT) scan after clinical decompensation. CT scan showing persistent colonic wall thickening over a 10 cm segment of transverse colon. This thickened segment of colon was previously contained in the Spigelian hernia from Figure 1. The white arrow shows the hernia which is now reduced in relation to Figure 1.

**Figure 4:**
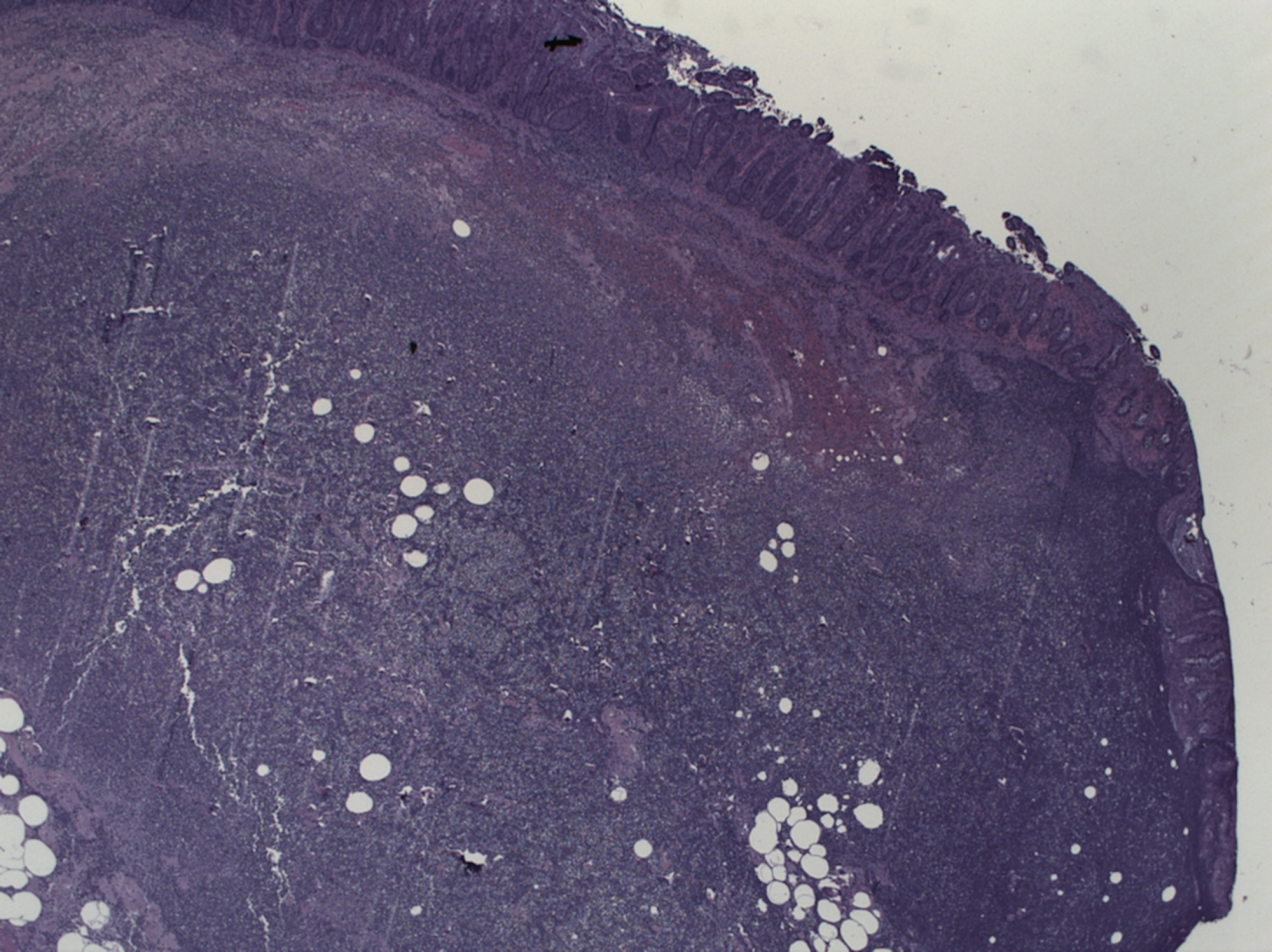
Pathology from resected colon showing surface erosion and friability.

**Figure 5:**
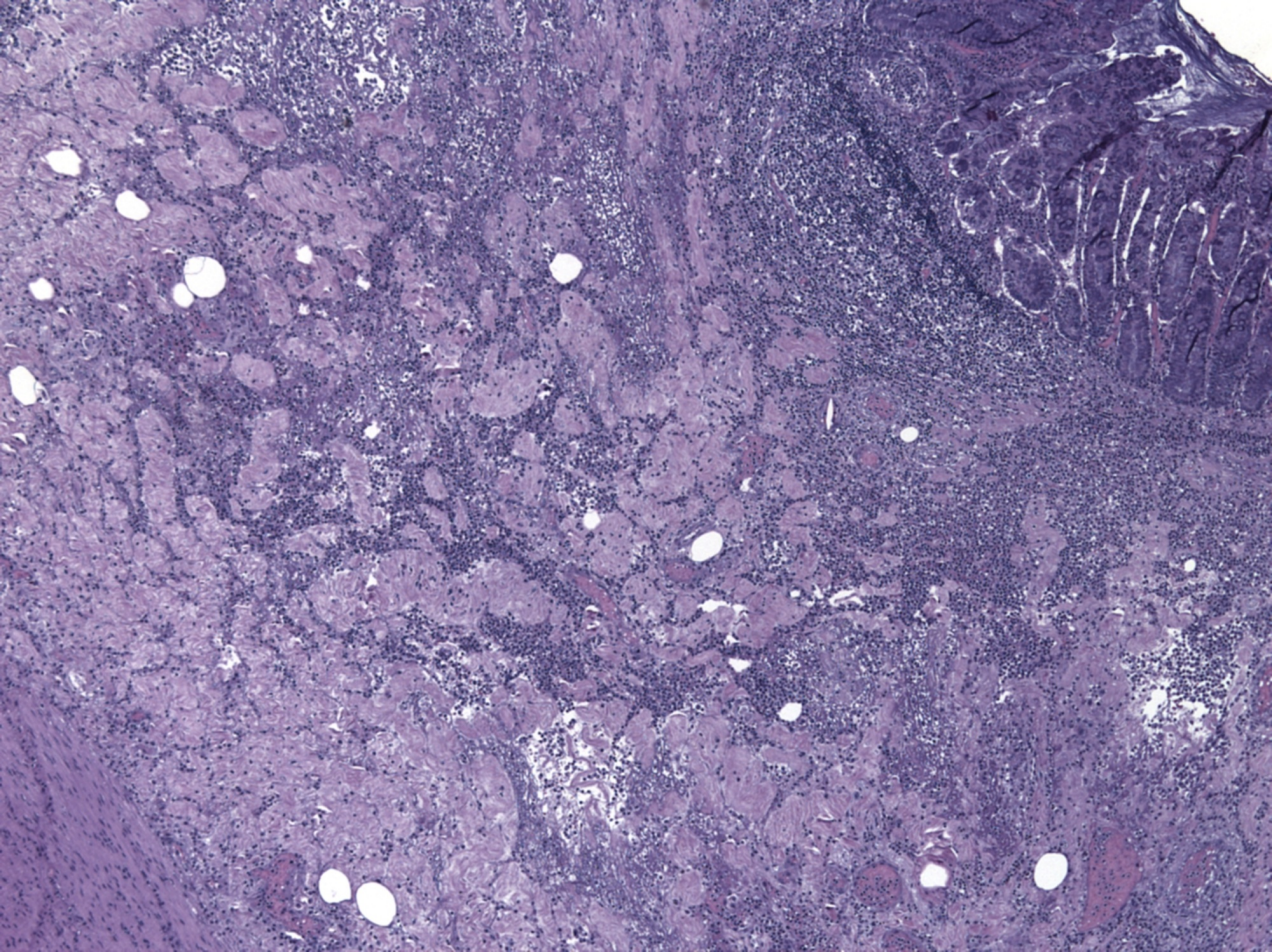
Pathology with transmural inflammatory cells.

**Figure 6:**
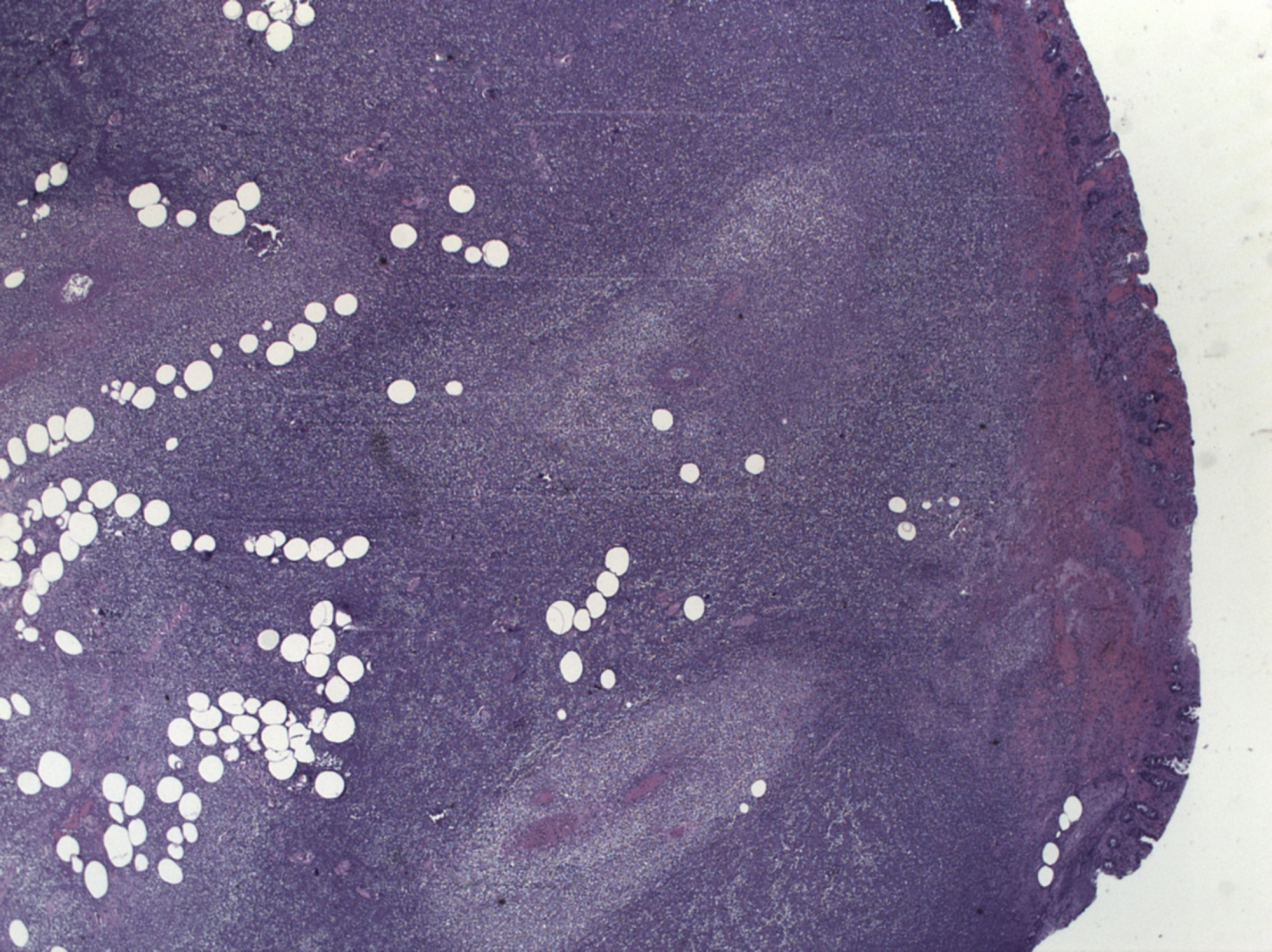
Pathology with spherical areas of necrosis representing abscess formation.

The patient was admitted and started on ceftriaxone and metronidazole for suspected microperforation to cover for intra-abdominal pathogens such as enterococci and anaerobic organisms. Surgery recommended antibiotic coverage and serial abdominal exams as the small abdominal hernia was found to be easily reducible. Over the next 24 hours, the patient’s abdominal pain improved initially, but he then developed worsening tachycardia with chills and hypoxia to 88% on a non-rebreather mask. The patient was intubated with subsequent hypotension requiring norepinephrine pressor support. A repeat CT abdomen, shown in Figure 3, showed persistent colonic wall thickening over a 10 cm segment of transverse colon. This thickened area was the segment of the colon that was previously contained in the Spigelian hernia on the first scan in Figure 1. The findings were concerning for ischemic colitis of the thickened segment and he was taken to the OR at that time for diagnostic laparoscopy, which was converted to an open procedure with resection of the thickened transverse colon segment and creation of an end colostomy. The pathology was consistent with ischemic colitis with mucosal ulceration and submucosal gangrenous necrosis. Figure 4 shows surface erosion while Figure 5 shows transmural inflammatory cells. These pathologic findings culminate in Figure 6 where circular areas of abscess are appreciated. The patient had a prolonged post-operative hospital course, but he eventually recovered and was extubated. He was discharged and underwent a laparoscopic takedown of his end colostomy with return to rectal function.

## Discussion

Spigelian hernias are very rare and comprise only 0.12% of abdominal wall hernias [1]. The name is credited to the discovering surgeon in Padua, Italy, Adriaan van den Spieghel [4]. The location of this hernia is along the lower abdomen adjacent to the semilunar line where there is no posterior sheath [1]. They are difficult to diagnose because they emanate from a small hernia ring defect, but are dangerous as they can easily become incarcerated. Adding to the diagnostic difficulty is the fact that there may not be a palpable lump, although the patient often presents with focal abdominal pain [1]. Imaging is usually needed for such a diagnosis and they are repaired most often using open mesh repair.

In reviewing the literature, there is only one reported case of a Spigelian hernia occurring after a colonoscopy and there are no cases reported that have occurred at the site of a prior LVAD placement, such as in this patient. This single case of Spigelian hernia after colonoscopy was presented by Demuro in 2012 [4]. There was a recently published article by Persinger and Basson in 2017, which details a Spigelian hernia preventing completion of a colonoscopy in a patient with a history of rectal cancer [3]. Our case is unique because the patient did not have a history of cancer and the Spigelian hernia did not prevent colonoscopy, as in the Persinger case. Quite to the contrary, the Spigelian hernia presented as a potential complication after colonoscopy. One must bear in mind that although the time-course suggests correlation, this does not mean that the colonoscopy was causative of the Spigelian hernia.

As patients live longer with more complicated histories, the incidence of unique presentations of procedural complications will only increase. Specifically, colonoscopy is a widely used tool with an estimated 15 million colonoscopies conducted in the United States in 2012 [2,5]. This number will continue to rise with the advent and implementation of quality healthcare measures, and as patients are encouraged to have screening colonoscopies after 50 years of age.

Our patient is unique due to his prior history of LVAD, but had a relatively common historical complaint of chronic diarrhea. He is like many other patients who have had prior procedures including cholecystectomy, appendectomy, or other intra-abdominal surgeries, which could predispose one to abdominal fascial plane weakening and increase the likelihood to developing an incarcerated hernia. The patient underwent a routine colonoscopy with no findings to suggest the patient may be susceptible to herniation. On presentation, he met SIRS criteria. This would certainly be concerning in the post-colonoscopy period for a microperforation, and he was treated appropriately. In the initial emergency department documentation there was no mention of possible hernia through the LVAD operative site, likely because this is a very uncommon diagnosis and microperforation is much more prevalent. This is where the availability bias enters the medical decision-making. Microperforation is clinically intuitive, but one must also be cognizant to avoid availability bias when a diagnosis is made based on the frequency that conditions have been seen by a clinician in the past [6]. It was only upon CT scan that the hernia was discovered and surgery found it to be reducible. It is interesting to note the follow-up CT scan report where radiologists mentioned that the colonic pathology was proximal to the area where the colon had herniated into the Spigelian sac. This is anatomically intuitive with the hernia interrupting the blood supply and causing subsequent necrosis of the intestine.

This case of Spigelian hernia incarceration is important for clinicians to acknowledge as it may be more common than is recognized and can have grave consequences.

## Conclusions

This case contributes to a clinician’s diagnostic repertoire and places emphasis on other less common post-colonoscopy complications that may occur. It also emphasizes that clinicians should avoid falling victim to availability bias. Availability bias often leads to clinicians not considering a diagnosis that is less common, and is well-explained in medical decision-making heuristic literature. In our case, avoiding availability bias by keeping a broad differential would lead clinicians to consider that this may not have been a microperforation, but could have been a rarer entity such as Spigelian hernia incarceration. By entertaining all of the possible diagnoses, medical complications can be minimized, and ultimately patient care will be improved.
